# Diagnostic sequence of chronic pain and severe mental illness: Relationship with mental health and hospitalization outcomes

**DOI:** 10.1177/20494637261451733

**Published:** 2026-05-28

**Authors:** Ruimin Ma, Eugenia Romano, Mark Ashworth, Nilufar Mossaheb, Kerem Böge, Davy Vancampfort, Robert Stewart, Brendon Stubbs

**Affiliations:** 1 Department of Psychological Medicine, Institute of Psychiatry, Psychology and Neuroscience (IoPPN), 4616 King’s College London, UK; 2 School of Life Course and Population Sciences, 4616 King’s College London, UK; 3 Division of Social Psychiatry, Department of Psychiatry and Psychotherapy, 27271 Medical University of Vienna Vienna, Austria; 4 Comprehensive Center for Clinical Neurosciences and Mental Health, 27271 Medical University of Vienna Vienna, Austria; 5 Department of Neuroscience and Psychiatry, Universitätsmedizin Berlin, corporate member of Freie Universität Berlin and Humboldt-Universität zu Berlin, Berlin, Germany; 6 Medical University Brandenburg-Theodor Fontane, Neuruppin, Germany; 7 German Center of Mental Health (DZPG), Berlin/Potsdam, Germany; 8 KU Leuven Department of Rehabilitation Sciences, Leuven, Belgium; 9 4958 University Psychiatric Centre KU Leuven, Leuven-Kortenberg, Belgium; 10 Leuven Brain Institute, Leuven, Belgium; 11 Mental Health for Older Adults and Dementia Clinical Academic Group, South London and Maudsley NHS Foundation Trust, London, UK

**Keywords:** chronic pain, severe mental illness, diagnostic sequence, mental health outcomes, hospital admissions

## Abstract

**Background:**

Chronic pain (CP) and severe mental illness (SMI) frequently co-occur, exacerbating clinical outcomes and healthcare burden. The influence of diagnostic sequence, that is, whether CP precedes SMI or vice versa, on mental health and hospitalization remains underexplored. This study investigates how the temporal order of CP and SMI diagnoses impacts illness burden, mental health comorbidities, and hospital admissions.

**Methods:**

Using linked electronic health records from primary care, mental health, and acute care in South London, we identified 1112 patients with comorbid CP and SMI (e.g. schizophrenia). Patients were grouped by diagnostic order: CP before SMI (CP-SMI, *n* = 413) versus SMI before CP (SMI-CP, *n* = 699). Outcomes included illness burden, mental health comorbidities, and hospital admissions, which were analysed via multivariable regression, adjusting for confounders.

**Results:**

Compared to the CP-SMI group, the SMI-CP group exhibited significantly lower odds of self-injury (OR = 0.55, 95% CI: 0.31–0.98), substance misuse (OR = 0.55, 95% CI: 0.34–0.86), depressive mood (OR = 0.63, 95% CI: 0.44–0.89), and depression diagnosis (OR = 0.58, 95% CI: 0.43–0.78). Among the SMI-CP group, older age reduced the odds of self-injury, substance misuse, and depressive mood but increased the risks of cognitive and physical problems. No significant differences were observed in psychiatric or general hospital admissions or physical comorbidities, though ethnic disparities persisted in psychiatric admissions.

**Conclusion:**

Patients diagnosed with CP before SMI experience worse mental health outcomes. This is likely due to delayed psychiatric intervention, therefore routine psychiatric screening aiming to improve outcomes and reduce healthcare burden at CP diagnosis should be evaluated.

## Introduction

Chronic pain (CP), defined as persistent or recurrent pain lasting over 3 months,^1^ affects approximately 25% of the global population, ranking among the leading causes of disability and healthcare utilization.^2^ Severe mental illness (SMI), conventionally encompassing schizophrenia, bipolar disorder, and major depressive disorder, imposes a significant burden, reducing life expectancy by 10–20 years due to physical comorbidities and impaired functional capacity.^3,4^ The co-occurrence of CP and SMI is common, with 25–50% of patients with SMI reporting CP, far exceeding general population rates.^5–7^ This comorbidity exacerbates clinical outcomes, including increased pain intensity, disability, and healthcare costs, while complicating treatment for both conditions.^8,9^

The clinical and public health significance of CP–SMI comorbidity is underscored by its impact on quality of life and healthcare systems. Individuals with both conditions face greater functional impairment, higher rates of self-injury, substance misuse, and depression, and increased reliance on acute care.^8,9^ Yet, pain management in SMI populations is often inadequate, with interventions extrapolated from general population studies and lacking tailoring to psychiatric needs.^8^ Integrated care models, combining psychiatric and pain management, are promising but under implemented, highlighting the need for evidence to guide clinical practice and policy.^10,11^

The relationship between CP and SMI is bidirectional, potentially driven by shared neurobiological, psychological, and social mechanisms. Neuroinflammatory pathways, hypothalamic–pituitary–adrenal axis dysregulation, and altered dopaminergic signalling contribute to both pain perception and psychiatric symptoms.^12–14^ Psychological factors, such as pain catastrophizing and emotional dysregulation, amplify pain sensitivity in SMI, while CP heightens psychological distress, potentially creating a vicious cycle.^15,16^ Socioeconomic disadvantages, including poverty and limited healthcare access, further exacerbate this comorbidity, disproportionately affecting marginalized populations.^17^ Despite these insights, significant gaps remain in understanding how the temporal sequence of CP and SMI diagnoses influences clinical trajectories and healthcare utilization.

Diagnostic sequence is a critical yet underexplored factor. CP may precede SMI, potentially mask psychiatric symptoms and delay diagnosis due to diagnostic overshadowing, where physical complaints dominate clinical attention.^18,19^ On the other hand, although SMI preceding CP may facilitate psychiatric management and mitigate CP-related psychological distress,^20^ this may also contribute to the delay CP diagnosis, as CP symptoms can be overshadowed by SMI and clinicians may attribute pain-related distress to the SMI instead.^18^ Altered pain perception in SMI, particularly schizophrenia, adds further complexity, with studies showing elevated pain thresholds that may delay detection of painful conditions.^21,22^ These patterns have profound implications, as delayed intervention for either condition can worsen outcomes, increase hospital admissions, and escalate costs, with CP in patients with SMI incurring an additional $5208 annually per patient.^23^

This study addresses these gaps by examining the impact of CP and SMI diagnostic sequence on clinical characteristics and hospital admission outcomes. Using linked electronic health record (EHR) data from primary care, mental health care, and acute care services in a South London catchment, we compare patients diagnosed with CP before SMI versus SMI before CP. By elucidating these patterns, this study aims to inform targeted interventions and integrated care strategies to improve outcomes and reduce healthcare burden in this underserved population.

## Methods

### Data sources

Data for this study were retrieved from a comprehensive population-based EHR linkage infrastructure, integrating routine data from three healthcare sectors: primary care (Lambeth Datanet; LDN), specialist mental healthcare (Clinical Record Interactive Search; CRIS), and acute care (Hospital Episode Statistics; HES). The CRIS infrastructure was developed by the NIHR Maudsley Biomedical Research Centre in collaboration with the South London and Maudsley NHS Foundation Trust (SLaM).^24^ SLaM provides all mental healthcare to an estimated 1.3 million residents in 4 South London boroughs (Lambeth, Southwark, Lewisham, and Croydon), providing researchers access to de-identified clinical data from the full record. Supplemented by over 100 natural language processing applications developed over the last 15 years, CRIS has supported over 400 peer-reviewed publications. CRIS has received full ethical approval for secondary data analysis (Oxford Research Ethics Committee C, reference 23/SC/0257).

CRIS data are linked to a range of other clinical data sources, including primary care record LDN, which encompasses 97% of GP services within the borough of Lambeth for over 827,000 registered adults. LDN provides pseudonymised structured clinical data from GP records, including sociodemographic information, consultation, service referrals, long-term physical conditions, and medication prescriptions.^25^ CRIS is also linked to HES, which provides England-wide data on hospital admission records, outpatient appointments, and emergency care.^26^

### Study population

Patients with comorbid SMI and CP, identified using ICD-10 codes (F2*, F30*, F31*, F32.3, and F33.3) for SMI diagnoses in CRIS and Read codes for CP in LDN^27^ (UK Read Codes, 2015), were eligible for inclusion. CP diagnoses were confirmed using either diagnostic Read codes or analgesic prescriptions lasting more than 90 days. This CP diagnostic criteria were developed by a multidisciplinary team comprising a physiotherapist, a GP, a psychiatrist and EHR researchers, and was further validated by a Patient and Public Involvement panel. CP diagnoses included in the current study encompassed a wide range of pain conditions, including abdominal, back, chest, facial, generalised, limb, and pelvic pain.

Patients with comorbid SMI and CP were also required to be aged 18 and above at time of their first SMI diagnosis and be active in both CRIS and LDN for at least 2 years from their index date (defined as the later of first SMI or CP diagnosis date) between 1st March 2011 and 31st March 2021. Eligible patients were classified into two case groups according to the temporal order of their SMI and CP diagnoses: (1) patients who received their SMI diagnosis prior to their CP diagnosis (SMI-CP group) and (2) those diagnosed with a CP before receiving an SMI diagnosis (CP-SMI group).

### Outcome measures

Co-primary outcomes comprised illness burden, mental disorder comorbidity, psychiatric, and general hospital admissions recorded within the window defined above. The Health of the National Outcome Scales (HoNOS) are routinely administered measures of illness burden in UK mental healthcare services.^28^ Individual HoNOS item scores first recorded after the index date, including clinical outcomes (behavioural disturbance, self-injury, problems with drinking and drugs, cognitive problems, physical illness, hallucinations and delusions, depressive mood, and other mental health problems) and social/functional outcomes (social relationships, daily living problems, living conditions problems, and occupational problems), were obtained from structured fields on the mental healthcare EHR via CRIS. Each HoNOS item is rated on a Likert Scale, ranging from 0 (i.e. no problem) to 4 (i.e. severe or very severe problem). Scores of 2 or above on individual HoNOS items were classified as having a problem and were included in the analyses as binary outcomes. A detailed description of the HoNOS is reported elsewhere.^29^ Mental disorder comorbidities ever being recorded, including diagnosis of depression, anxiety, alcohol and substance misuse, dementia, learning disability, eating disorder and personality disorder, were ascertained from LDN using Read codes. Psychiatric and general hospital admissions, ascertained from HES, were incorporated into the analysis both as binary variables (presence or absence of admission) and as continuous variables (total number of admissions per individual). Physical comorbidities as a secondary outcome were ascertained from LDN. Date of diagnosis for 17 physical comorbidities (including myocardial infarct, HIV/AIDS, renal failure, respiratory disease, connective tissue disorders, peptic ulcer disease, heart failure, mild and severe liver disease, stroke, paralytic syndromes, dementia, diabetes and associated complications, cancer, peripheral arterial disease, and Metabases) were used to calculate both the number of physical comorbidities recorded after the index date and the total number of physical comorbidities ever recorded.

Covariates, including age at the index date, gender, ethnicity, and 2011-defined lower super output area (LSOA), were obtained. Ethnicity was categorised into 5 categories (White, Black, Asian, Mixed, and Others). LSOAs are geographic areas of residence (32,8444 in total), each containing about 1500 residents or 650 households in England.^30^ A deprivation score, the Index of Multiple Deprivation (IMD15), was calculated based on areas of residence closest to patients’ index date.

### Statistical analysis

Differences in characteristics between the SMI-CP and CP-SMI cohorts were investigated using t-tests (for continuous variables) and Pearson’s Chi-Square tests (for categorical variables). Multivariable linear and logistic regression models were used to assess the relationship between two case groups and continuous variables (i.e. physical comorbidities, number of psychiatric and general hospital admissions) and categorical variables (i.e. HoNOS scores and mental health comorbidity), respectively. Analyses were both unadjusted and adjusted for age at the index date, ethnicity, deprivation score, SMI diagnoses, CP diagnoses, and the number of physical comorbidities ever recorded. One CP diagnosis, chronic regional pain syndrome/vertebral pain, was excluded from all analyses due to the small number of patients assigned to the group (*n* = 2, 0.2%). Results are reported as coefficients for linear regression analyses and odds ratios (ORs) for logistic regression analyses, each with corresponding 95% confidence intervals (CIs). Model fit was assessed using F-statistics and adjusted R^2^ for linear regression models, and likelihood ratio chi-square (LR χ^2^) statistics with degrees of freedom and pseudo-R^2^ for logistic regression models. All analyses were conducted using STATA 18 (Stata Corp LP, College Station, TX).

## Results

### Sample characteristics

The cohort included 1112 patients: 413 with CP-SMI, and 699 with SMI-CP. Mean age was 60.2 years (SD = 14.9), with 55.2% female. No significant differences were observed in age, gender, ethnicity, deprivation, or SMI diagnoses. The CP-SMI group had higher proportions of back (13.8% vs 8.1%, *p* = .003), facial (7.4% vs 3.9%, *p* = .01), and upper limb pain (5.0% vs 2.2%, *p* = .02).

On HoNOS, the CP-SMI group exhibited higher rates of self-injury (11.9% vs 6.5%, *p* = .01), substance misuse (19.7% vs 13.2%, *p* = .01), depressive mood (43.9% vs 32.7%, *p* < .01), and other mental health problems (62.9% vs 55.2%, *p* = .03). Depression diagnoses were more prevalent in the CP-SMI group (62.7% vs 51.5%, *p* < .01) (Table 1).

**Table 1.** Comparison of baseline characteristics between CP-SMI and SMI-CP groups.

Variables	Total (*N* = 1112)	CP-SMI group (*N* = 413)	SMI-CP group (*N* = 699)		
	Mean (SD) or %	Mean (SD) or %	Mean (SD) or %	t-value or chi-square (df)	*p* value
Age	60.17 (14.85)	59.69 (14.92)	60.45 (14.82)	−0.83 (1110)	0.41
Age at index date	51.06 (15.42)	50.84 (0.76)	51.19 (15.42)	−0.37 (1110)	0.71
Gender	​			0.99 (1)	0.32
Male	44.78	42.86	45.92	​	​
Female	55.22	57.14	54.08	​	​
Ethnicity	​			6.26 (4)	0.18
White	49.41	52.78	47.42	​	​
Black	35.28	31.72	37.39	​	​
Asian	6.03	5.33	6.45	​	​
Mixed	5.67	5.57	5.73	​	​
Others	3.60	4.60	3.01	​	​
Deprivation score	31.21 (9.26)	31.40 (9.35)	31.10 (9.21)	0.52 (1110)	0.60
SMI diagnosis	​			4.26 (3)	​
Schizophrenia	64.12	65.86	63.09	​	0.23
Bipolar	22.48	20.58	23.61	​	​
Depressive	7.91	9.20	7.15	​	​
Unspecified	5.49	4.36	6.15	​	​
CP diagnosis	​			21.29 (8)	**0.01**
Abdominal pain	12.30	11.47	12.82	​	​
Back pain	10.27	13.82	8.06	​	**0.003**
Chest pain	8.13	7.94	8.24	​	​
Facial pain	5.19	7.35	3.85	​	**0.01**
Generalised pain not localised/Pain unspecified	37.47	34.12	39.56	​	​
Lower limb pain	18.96	16.18	20.70	​	​
Chronic regional pain syndrome/Vertebral pain	0.23	0.29	0.18	​	​
Pelvic pain	4.18	3.82	4.40	​	​
Upper limb pain	3.27	5.00	2.20	​	**0.02**
HONOS scale	​			​	
Behavioural disturbance	20.88	22.19	20.08	0.52 (1)	0.47
Self-injury	8.55	11.90	6.50	7.20 (1)	**0.01**
Problem drinking drugs	15.69	19.68	13.24	6.02 (1)	**0.01**
Cognitive problems	18.58	16.72	19.72	1.15 (1)	0.28
Hallucinations delusions	41.64	44.52	39.88	1.70 (1)	0.19
Depressive mood	36.96	43.87	32.74	10.23 (1)	**<0.01**
Physical illness	45.85	48.55	44.20	1.47 (1)	0.23
Social relationships	33.82	35.05	33.07	0.34 (1)	0.56
Daily living	18.11	18.42	17.93	0.03 (1)	0.86
Living conditions	34.19	32.00	34.98	0.37 (1)	0.54
Work	33.62	32.69	34.19	0.20 (1)	0.66
Other mental health problems	58.14	62.90	55.23	4.66 (1)	**0.03**
Psychiatric admissions after index	17.81	18.40	17.45	0.16 (1)	0.69
Number of psychiatric admissions after index	0.63 (2.08)	0.61 (2.11)	0.65 (2.07)	−0.32 (1110)	0.75
General admissions after index	41.82	41.40	42.06	0.05 (1)	0.83
Number of general admissions after index	2.62 (20.87)	2.87 (17.38)	2.47 (22.69)	0.31 (1110)	0.76
Numbers of physical comorbidities ever	0.83 (0.97)	0.82 (0.95)	0.84 (0.97)	−0.26 (1110)	0.79
Number of physical comorbidities before index	0.59 (0.84)	0.60 (0.86)	0.59 (0.84)	0.17 (1110)	0.86
Number of physical comorbidities after index	0.24 (0.50)	0.23 (0.48)	0.25 (0.51)	−0.81 (1110)	0.42
Depression diagnosis ever	55.67	62.71	51.50	13.22 (1)	**<0.01**
Anxiety diagnosis ever	47.12	49.64	45.64	1.67 (1)	0.20
Alcohol misuse diagnosis ever	40.29	36.56	42.49	3.79 (1)	0.05
Dementia diagnosis ever	4.14	4.12	4.15	<0.01 (1)	0.98
Eating disorder diagnosis ever	1.44	1.21	1.57	0.24 (1)	0.62
Substance misuse diagnosis ever	23.65	25.91	22.32	1.85 (1)	0.17
Learning disability diagnosis ever	2.25	1.21	2.86	3.22 (1)	0.07
Personality disorder diagnosis ever	10.43	10.41	10.44	<0.01 (1)	0.99

Variables with a *p*-value <.05 are marked in bold.

Abbreviations: CP: Chronic pain, N: number, SD: standard deviation, SMI: Severe mental illness.

### Clinical and functional outcomes

After adjustment, the SMI-CP group had lower odds of self-injury (OR = 0.55, 95% CI: 0.31–0.98), substance misuse (OR = 0.55, 95% CI: 0.34–0.86), and depressive mood (OR = 0.63, 95% CI: 0.44–0.89) compared to the CP-SMI group (Table 2). Older age was protective against self-injury (OR = 0.98, 95% CI: 0.95–0.99), substance misuse (OR = 0.97, 95% CI: 0.95–0.99), and depressive mood (OR = 0.98, 95% CI: 0.97–0.99), but increased risks of cognitive (OR = 1.02, 95% CI: 1.00–1.04) and physical problems (OR = 1.03, 95% CI: 1.02–1.05). No significant differences were observed in HoNOS social/functional outcomes (Table 3).

**Table 2.** Multivariable logistic regression models for HoNOS clinical outcomes, controlling for covariates.

Predictors	HoNOS items – clinical outcomes							​
	Behavioural disturbance OR (95% CI)	Self-injury OR (95% CI)	Problem drinking drug OR (95% CI)	Cognitive problems OR (95% CI)	Hallucinations delusions OR (95% CI)	Depressive mood OR (95% CI)	Physical illness OR (95% CI)	Other mental health problems OR (95% CI)
Cohorts	​							​
CP-SMI group	Reference	Reference	Reference	Reference	Reference	Reference	Reference	Reference
SMI-CP group	0.84 (0.56, 1.25)	**0.55 (0.31,0.98)**	**0.55 (0.34, 0.86)**	1.41 (0.90, 2.20)	0.93 (0.67, 1.31)	**0.63 (0.44, 0.89)**	0.76 (0.53, 1.09)	0.80 (0.57, 1.12)
SMI diagnosis	​							​
Schizophrenia	Reference	Reference	Reference	Reference	Reference	Reference	Reference	Reference
Bipolar disorder	1.11 (0.70, 1.77)	1.51 (0.77, 2.96)	1.57 (0.92, 2.70)	0.82 (0.48, 1.39)	**0.28 (0.18, 0.44)**	**1.86 (0.97, 0.99)**	0.97 (0.62, 1.49)	1.03 (0.68, 1.54)
Depressive	**0.30 (0.10, 0.88)**	**3.05 (1.27, 7.35)**	1.02 (0.40, 2.59)	0.95 (0.42, 2.17)	0.84 (0.46, 1.55)	**5.58 (2.84, 10.98)**	1.56 (0.81, 3.01)	1.22 (0.65, 2.31)
Unspecified SMI	0.91 (0.38, 2.22)	2.31 (0.73, 7.34)	1.42 (0.50, 4.06)	0.70 (0.26, 1.92)	0.73 (0.36, 1.51)	1.54 (0.72, 3.29)	1.14 (0.51, 2.52)	1.15 (0.55, 2.39)
Age at index date	0.99 (0.98, 1.01)	**0.98 (0.95, 0.99)**	**0.97 (0.95, 0.99)**	**1.02 (1.00, 1.04)**	1.01 (0.99, 1.02)	**0.98 (0.97, 0.99)**	**1.03 (1.02, 1.05)**	**0.98 (0.97, 0.99)**
Gender	​							​
Male	Reference	Reference	Reference	Reference	Reference	Reference	Reference	Reference
Female	0.99 (0.66, 1.47)	1.06 (0.59, 1.90)	**0.29 (0.18, 0.46)**	0.97 (0.64, 1.49)	0.94 (0.67, 1.30)	1.34 (0.95, 1.92)	0.88 (0.62, 1.26)	1.12 (0.81, 1.56)
Ethnicity	​							​
White	Reference	Reference	Reference	Reference	Reference	Reference	Reference	Reference
Black	0.84 (0.54, 1.32)	0.52 (0.26, 1.02)	**0.53 (0.31, 0.90)**	0.71 (0.43, 1.17)	1.20 (0.83, 1.74)	**0.59 (0.39, 0.87)**	0.76 (0.51, 1.14)	**0.58 (0.40, 0.84)**
Asian	0.76 (0.32, 1.78)	0.44 (0.10, 1.95)	0.31 (0.09, 1.10)	0.64 (0.25, 1.65)	0.91 (0.45, 1.83)	**0.43 (0.19, 0.94)**	0.47 (0.21, 1.05)	0.72 (0.36, 1.43)
Mixed	0.58 (0.25, 1.39)	0.32 (0.72, 1.43)	0.71 (0.28, 1.75)	1.08 (0.49, 2.36)	0.90 (0.47, 1.71)	0.58 (0.29, 1.16)	0.50 (0.25, 1.01)	0.64 (0.34, 1.21)
Other	1.61 (0.57, 4.50)	0.81 (0.21, 3.10)	1.44 (0.50, 4.18)	0.49 (0.11, 2.25)	0.95 (0.36, 2.51)	1.35 (0.50, 3.66)	1.01 (0.37, 2.74)	1.20 (0.44, 3.29)
Deprivation score	0.98 (0.96, 1.00)	0.99 (0.97, 1.03)	**1.03 (1.00, 1.06)**	1.00 (0.98, 1.03)	1.00 (0.99, 1.02)	0.99 (0.98, 1.02)	1.02 (0.99, 1.04)	0.99 (0.98, 1.01)
CP diagnosis	​							​
Abdominal pain	Reference	Reference	Reference	Reference	Reference	Reference	Reference	Reference
Back pain	0.70 (0.30, 1.68)	1.33 (0.40, 4.45)	0.56 (0.20, 1.57)	1.32 (0.47, 3.73)	0.61 (0.30, 1.22)	0.71 (0.34, 1.47)	0.82 (0.38, 1.73)	0.76 (0.38, 1.54)
Chest pain	0.95 (0.41, 2.23)	1.90 (0.58, 6.24)	0.65 (0.24, 1.78)	1.43 (0.49, 4.14)	1.03 (0.51, 2.11)	0.89 (0.43, 1.86)	0.97 (0.45, 2.09)	1.05 (0.51, 2.17)
Facial pain	1.41 (0.56, 3.57)	1.60 (0.41, 6.32)	1.50 (0.52, 4.37)	2.36 (0.77, 7.25)	0.93 (0.40, 2.19)	0.90 (0.38, 2.12)	1.13 (0.47, 2.76)	1.52 (0.61, 3.77)
Generalised pain not localised/Pain unspecified	1.01 (0.53, 1.92)	1.50 (0.55, 4.03)	1.19 (0.57, 2.50)	1.75 (0.77, 3.97)	0.62 (0.36, 1.08)	0.62 (0.35, 1.09)	0.71 (0.39, 1.29)	0.83 (0.48, 1.43)
Lower limb pain	0.83 (0.40, 1.72)	1.40 (0.47, 4.21)	0.93 (0.40, 2.13)	1.51 (0.62, 3.63)	0.75 (0.42, 1.36)	0.93 (0.51, 1.72)	0.92 (0.48, 1.75)	0.98 (0.54, 1.78)
Pelvic pain	0.94 (0.30, 2.96)	0.50 (0.06, 4.50)	1.19 (0.33, 4.26)	1.39 (0.33, 5.78)	0.40 (0.14, 1.15)	0.61 (0.22, 1.65)	1.21 (0.43, 3.36)	0.44 (0.17, 1.15)
Upper limb pain	2.57 (0.92, 7.21)	1.15 (0.20, 6.53)	1.15 (0.30, 4.34)	2.54 (0.71, 9.07)	0.92 (0.35, 2.43)	1.07 (0.39, 2.97)	0.39 (0.12, 1.30)	0.95 (0.35, 2.55)
Number of physical comorbidities ever	0.99 (0.77, 1.27)	0.86 (0.57, 1.28)	0.86 (0.30, 4.34)	1.08 (0.85, 1.38)	0.90 (0.74, 1.11)	0.98 (0.79, 1.22)	**1.90 (1.53, 2.37)**	0.89 (0.73, 1.08)
LR χ^2^ (df)	24.43 (19)	**30.18 (19)**	**69.00 (19)**	23.6 (19)	**51.52 (19)**	**82.16 (19)**	**132.74 (19)**	**38.99 (19)**
Pseudo-R^2^	0.04	0.08	0.12	0.04	0.06	0.10	0.15	0.04

Variables with a *p*-value <.05 are marked in bold.

Abbreviations: CI: Confidence interval, CP: Chronic pain, df: degrees of freedom, HoNos: The Health of the National Outcome Scales, OR: odds ratio, SMI: Severe mental illness.

**Table 3.** Multivariable logistic regression models for HoNOS social and functional outcomes, controlling for covariates.

Predictors	HoNOS items – social and functional outcomes			
	Social relationships OR (95% CI)	Daily living OR (95% CI)	Living conditions OR (95% CI)	Work OR (95% CI)
Cohorts	​			
CP-SMI group	Reference	Reference	Reference	Reference
SMI-CP group	0.94 (0.66, 1.32)	1.07 (0.71, 1.61)	1.07 (0.75, 1.51)	1.05 (0.74,1.48)
SMI diagnosis	​			
Schizophrenia	Reference	Reference	Reference	Reference
Bipolar	1.07 (0.71, 1.61)	0.75 (0.44, 1.25)	0.97 (0.64, 1.47)	0.95 (0.63, 1.45)
Depressive	0.78 (0.40, 1.54)	0.65 (0.27, 1.52)	0.65 (0.32, 1.31)	1.12 (0.59, 2.13)
Unspecified SMI	1.08 (0.51, 2.30)	0.72 (0.28, 1.83)	1.01 (0.48, 2.14)	1.26 (0.60, 2.64)
Age at index date	**0.98 (0.97, 0.99)**	0.99 (0.98, 1.02)	**1.02 (1.00, 1.03)**	1.00 (0.99, 1.02)
Gender	​			
Male	Reference	Reference	Reference	Reference
Female	1.19 (0.84, 1.67)	0.98 (0.65, 1.47)	1.04 (0.74, 1.47)	0.81 (0.58, 1.14)
Ethnicity	​			
White	Reference	Reference	Reference	Reference
Black	0.95 (0.64, 1.39)	1.27 (0.80, 2.01)	0.90 (0.61, 1.34)	0.89 (0.61, 1.31)
Asian	1.12 (0.55, 2.27)	0.84 (0.35, 2.06)	0.62 (0.28, 1.35)	0.51 (0.23, 1.12)
Mixed	0.71 (0.36, 1.41)	1.15 (0.53, 2.52)	1.09 (0.57, 2.08)	0.52 (0.25, 1.05)
Other	0.74 (0.27, 2.03)	1.23 (0.38, 3.91)	0.99 (0.38, 2.63)	0.49 (0.17, 1.42)
Deprivation score	0.99 (0.97, 1.01)	0.99 (0.96, 1.01)	0.99 (0.97, 1.01)	1.00 (0.98, 1.02)
CP diagnosis	​			
Abdominal pain	Reference	Reference	Reference	Reference
Back pain	1.42 (0.71, 2.86)	0.97 (0.38, 2.47)	0.51 (0.24, 1.07)	**0.45 (0.21, 0.96)**
Chest pain	0.88 (0.42, 1.84)	1.73 (0.72, 4.13)	1.23 (0.60, 2.49)	1.24 (0.61, 2.51)
Facial pain	1.30 (0.56, 3.00)	1.76 (0.64, 4.87)	0.51 (0.20, 1.29)	0.98 (0.42, 2.27)
Generalised pain not localised/Pain unspecified	1.02 (0.58, 1.77)	1.18 (0.57, 2.43)	0.78 (0.45, 1.35)	0.75 (0.43, 1.31)
Lower limb pain	1.28 (0.70, 2.34)	1.79 (0.85, 3.80)	0.74 (0.41, 1.36)	0.79 (0.43, 1.44)
Pelvic pain	0.44 (0.15, 1.33)	0.52 (0.11, 2.55)	**0.16 (0.04, 0.75)**	0.54 (0.19, 1.54)
Upper limb pain	1.01 (0.37, 2.76)	2.02 (0.64, 6.32)	0.96 (0.35, 2.60)	0.58 (0.20, 1.67)
Number of physical comorbidities ever	0.91 (0.74, 1.13)	1.05 (0.82, 1.34)	0.96 (0.78, 1.18)	1.02 (0.83, 1.25)
LR χ^2^ (df)	25.15 (19)	13.57 (19)	25.95 (19)	18.14 (19)
Pseudo-R^2^	0.03	0.02	0.03	0.02

Variables with a *p*-value <.05 are marked in bold.

Abbreviations: CI: Confidence interval, CP: Chronic pain, df: degrees of freedom, HoNos: The Health of the National Outcome Scales, OR: odds ratio, SMI: Severe mental illness.

### Mental health comorbidities

The SMI-CP group had lower odds of depression diagnosis (OR = 0.58, 95% CI: 0.43–0.78) (Table 4). Bipolar (OR = 2.15, 95% CI: 1.50–3.06) and depressive disorders (OR = 36.77, 95% CI: 8.84–152.87) increased the odds of depression diagnosis compared to schizophrenia. Female gender was associated with higher odds of depression (OR = 1.41, 95% CI: 1.06–1.89) and anxiety (OR = 1.56, 95% CI: 1.17–2.08), but lower odds of substance misuse (OR = 0.57, 95% CI: 0.41–0.81).

**Table 4.** Multivariable logistic regression models for other mental health comorbidities, controlling for covariates.

Predictors	Mental health disorders ever developed							
	Depression OR (95% CI)	Anxiety OR (95% CI)	Alcohol misuse OR (95% CI)	Dementia OR (95% CI)	Substance misuse OR (95% CI)	Learning disability OR (95% CI)	Eating disorder OR (95% CI)	Personality disorder OR (95% CI)
Cohorts								
CP-SMI group	Reference	Reference	Reference	Reference	Reference	Reference	Reference	Reference
SMI-CP group	**0.58 (0.43, 0.78)**	0.87 (0.65, 1.16)	1.23 (0.92, 1.65)	0.79 (0.32, 1.98)	0.74 (0.52, 1.04)	2.80 (0.91, 8.62)	0.94 (0.28, 3.20)	1.08 (0.67, 1.75)
SMI diagnosis								
Schizophrenia	Reference	Reference	Reference	Reference	Reference	Reference	Reference	Reference
Bipolar	**2.15 (1.50, 3.06)**	1.17 (0.83, 1.66)	0.95 (0.67, 1.35)	1.41 (0.47, 4.28)	**1.61 (1.07, 2.40)**	0.51 (0.14, 1.87)	1.69 (0.45, 6.36)	2.04 (1.22, 3.40)
Depressive	**36.77 (8.84, 152.87)**	**2.56 (1.46, 4.49)**	0.57 (0.32, 1.01)	1.00 (0.19, 5.25)	1.18 (0.60, 2.30)	0.56 (0.07, 4.51)	1.33 (0.14, 12.57)	1.13 (0.42, 3.05)
Unspecified SMI	**2.01 (1.07, 3.80)**	1.09 (0.57, 2.06)	0.89 (0.46, 1.69)	1.51 (0.16, 13.97)	1.49 (0.70, 3.20)	Omitted	2.84 (0.28, 28.31)	1.23 (0.41, 3.71)
Age at index date	**0.98 (0.97, 0.99)**	**0.98 (0.97, 0.99)**	1.00 (0.99, 1.01)	**1.15 (1.10, 1.20)**	**0.98 (0.96, 0.99)**	0.97 (0.94, 1.01)	0.99 (0.95, 1.03)	0.99 (0.97, 1.01)
Gender								
Male	Reference	Reference	Reference	Reference	Reference	Reference	Reference	Reference
Female	**1.41 (1.06, 1.89)**	**1.56 (1.17, 2.08)**	0.76 (0.57, 1.01)	1.13 (0.44, 2.85)	**0.57 (0.41, 0.81)**	0.99 (0.40, 2.47)	**9.31 (1.16, 74.46)**	0.71 (0.44, 1.13)
Ethnicity								
White	Reference	Reference	Reference	Reference	Reference	Reference	Reference	Reference
Black	**0.64 (0.46, 0.89)**	**0.48 (0.35, 0.66)**	0.79 (0.57, 1.09)	1.06 (0.36, 3.18)	**0.64 (0.43, 0.95)**	1.59 (0.57, 4.42)	0.12 (0.01, 1.01)	**0.35 (0.19, 0.65)**
Asian	1.07 (0.59, 1.93)	**0.43 (0.24, 0.78)**	0.99 (0.55, 1.76)	1.68 (0.37, 7.71)	0.61 (0.28, 1.31)	1.45 (0.28, 7.53)	0.78 (0.09, 6.65)	0.55 (0.19, 1.62)
Mixed	1.02 (0.56, 1.89)	0.83 (0.45, 1.50)	0.88 (0.47, 1.60)	5.21 (1.13, 24.07)	1.21 (0.62, 2.34)	0.74 (0.08, 6.52)	Omitted	1.07 (0.45, 2.55)
Other	0.94 (0.42, 2.13)	**0.39 (0.18, 0.86)**	0.78 (0.36, 1.69)	Omitted	0.46 (0.17, 1.26)	Omitted	Omitted	0.83 (0.27, 2.53)
Deprivation score	1.01 (0.99, 1.02)	0.99 (0.98, 1.01)	1.01 (0.99, 1.02)	**1.05 (1.00, 1.11)**	1.01 (0.99, 1.03)	0.96 (0.91, 1.01)	1.01 (0.95, 1.08)	1.00 (0.98, 1.03)
CP diagnosis								
Abdominal pain	Reference	Reference	Reference	Reference	Reference	Reference	Reference	Reference
Back pain	0.67 (0.36, 1.24)	0.68 (0.37, 1.23)	0.69 (0.37, 1.27)	0.33 (0.02, 4.66)	1.16 (0.58, 2.33)	0.64 (0.11, 3.80)	0.49 (0.03, 9.01)	0.66 (0.24, 1.82)
Chest pain	1.08 (0.57, 2.05)	1.37 (0.73, 2.57)	0.86 (0.46, 1.61)	1.18 (0.08, 15.92)	0.80 (0.37, 1.71)	1.11 (0.23, 5.33)	0.80 (0.04, 14.59)	0.95 (0.36, 2.55)
Facial pain	1.02 (0.48, 2.18)	1.29 (0.62, 2.70)	0.65 (0.31, 1.40)	Omitted	0.93 (0.39, 2.23)	0.74 (0.08, 7.15)	0.66 (0.04, 11.60)	1.03 (0.35, 3.08)
Generalised pain not localised/Pain unspecified	0.81 (0.50, 1.31)	0.84 (0.53, 1.33)	1.18 (0.74, 1.86)	1.09 (0.21, 5.79)	1.02 (0.59, 1.78)	0.71 (0.19, 2.55)	0.76 (0.08, 7.29)	0.96 (0.46, 2.00)
Lower limb pain	0.87 (0.52, 1.47)	0.94 (0.57, 1.57)	1.19 (0.72, 1.96)	0.68 (0.99, 4.62)	1.41 (0.78, 2.57)	0.33 (0.06, 1.90)	0.96 (0.09, 10.48)	0.92 (0.41, 2.10)
Pelvic pain	1.69 (0.74, 3.90)	2.21 (0.97, 5.03)	0.80 (0.36, 1.79)	1.38 (0.10, 20.17)	1.05 (0.41, 2.66)	Omitted	Omitted	0.77 (0.20, 3.01)
Upper limb pain	1.01 (0.41, 2.50)	0.73 (0.30, 1.76)	0.40 (0.15, 1.08)	2.84 (0.33, 24.60)	0.79 (0.26, 2.38)	1.22 (0.12, 12.49)	Omitted	0.70 (0.14, 3.43)
Number of physical comorbidities ever	1.19 (0.99, 1.41)	**0.84 (0.70, 0.99)**	**1.24 (1.05, 1.47)**	0.90 (0.59, 1.36)	0.86 (0.69, 1.09)	1.40 (0.83, 2.36)	0.67 (0.27, 1.68)	0.84 (0.61, 1.15)
LR χ^2^ (df)	**133.71 (19)**	**91.24 (19)**	**38.71 (19)**	**85.57 (19)**	**56.31 (19)**	15.68 (19)	18.69 (19)	**36.69 (19)**
Pseudo-R^2^	0.11	0.07	0.03	0.35	0.06	0.08	0.16	0.07

Variables with a *p*-value <.05 are marked in bold.

Abbreviations: CI: Confidence interval, CP: Chronic pain, df: degrees of freedom, HoNos: The Health of the National Outcome Scales, OR: odds ratio, SMI: Severe mental illness.

### Hospital admissions and physical comorbidities

No significant differences in psychiatric or general hospital admissions were observed between groups (Table 5). Older age reduced psychiatric admission odds (OR = 0.98, 95% CI: 0.96–0.99) and frequency (coefficient = −0.01, 95% CI: −0.03–0.00). Black (OR = 1.82, 95% CI: 1.21–2.74) and Mixed ethnicity (OR = 2.27, 95% CI: 1.15–4.48) increased psychiatric admission odds. Lower general admission odds were noted for back (OR = 0.45, 95% CI: 0.25–0.82), chest, and lower limb pain. Physical comorbidity counts were similar across groups (Supplementary Table 1).

**Table 5.** Multivariable linear and logistic regression models for hospital admissions, controlling for covariates.

Predictors	Hospital admissions			
	Psychiatric admissions OR (95% CI)^a^	Number of psychiatric admissions coefficient (95% CI)^b^	General admissions OR (95% CI)^a^	Number of general admissions coefficient (95% CI)^b^
Cohorts	​			
CP-SMI group	Reference	Reference	Reference	Reference
SMI-CP group	0.99 (0.68, 1.43)	0.06 (−0.24, 0.35)	1.04 (0.78, 1.39)	−0.08 (−3.13, 2.96)
SMI diagnosis	​			
Schizophrenia	Reference	Reference	Reference	Reference
Bipolar	1.39 (0.91, 2.12)	0.31 (−0.05, 0.66)	1.08 (0.76, 1.54)	−0.81 (−4.49, 2.87)
Depressive	**0.29 (0.10, 0.84)**	−0.41 (−0.95, 0.14)	**1.80 (1.06, 3.08)**	−0.71 (−6.37, 4.95)
Unspecified SMI	0.55 (0.21, 1.45)	−0.42 (−1.07, 0.23)	0.95 (0.50, 1.82)	−1.55 (−8.30, 5.19)
Age at index date	**0.98 (0.96, 0.99)**	**−0.01 (-0.03, -0.00)**	0.99 (0.98, 1.01)	−0.03 (−0.14, 0.09)
Gender	​			
Male	Reference	Reference	Reference	Reference
Female	0.99 (0.70, 1.43)	−0.13 (−0.42, 0.16)	1.08 (0.81, 1.43)	−1.64 (−4.63, 1.35)
Ethnicity	​			
White	Reference	Reference	Reference	Reference
Black	**1.82 (1.21, 2.74)**	**0.44 (0.11, 0.76)**	0.81 (0.59, 1.12)	1.97 (−1.40, 5.35)
Asian	0.68 (0.28, 1.70)	0.15 (−0.44, 0.74)	0.86 (0.48, 1.54)	−1.17 (−7.24, 4.91)
Mixed	**2.27 (1.15, 4.48)**	**0.89 (0.29, 1.50)**	0.97 (0.54, 1.76)	−1.49 (−7.79, 4.81)
Other	1.15 (0.42, 3.21)	0.35 (−0.42, 1.13)	2.26 (1.06, 4.84)	−0.84 (−8.83, 7.15)
Deprivation score	0.99 (0.97, 1.01)	−0.01 (−0.02, 0.01)	1.00 (0.98, 1.02)	−0.00 (−0.16, 0.16)
CP diagnosis	​			
Abdominal pain	Reference	Reference	Reference	Reference
Back pain	0.59 (0.26, 1.32)	−0.11 (−0.71, 0.50)	**0.45 (0.25, 0.82)**	−5.42 (−11.65, 0.81)
Chest pain	0.92 (0.44, 1.95)	0.18 (−0.46, 0.81)	**0.43 (0.23, 0.81)**	−2.68 (−9.25, 3.89)
Facial pain	1.09 (0.46, 2.58)	0.05 (−0.68, 0.79)	0.50 (0.24, 1.03)	−5.80 (−13.43, 1.82)
Generalised pain not localised/Pain unspecified	0.91 (0.53, 1.60)	0.17 (−0.30, 0.64)	**0.50 (0.32, 0.79)**	**−5.81 (-10.66, -0.96)**
Lower limb pain	1.03 (0.56, 1.91)	0.29 (−0.22, 0.81)	**0.53 (0.32, 0.87)**	**−6.40 (-11.75, -1.05)**
Pelvic pain	0.97 (0.38, 2.47)	0.19 (−0.61, 0.98)	1.41 (0.65, 3.04)	−4.39 (−12.59, 3.82)
Upper limb pain	0.51 (0.14, 1.90)	−0.32 (−1.19, 0.56)	0.48 (0.20, 1.15)	−6.17 (−15.24, 2.91)
Number of physical comorbidities ever	1.02 (0.81, 1.29)	−0.07 (−0.25, 0.10)	**1.48 (1.24, 1.75)**	**2.68 (0.88, 4.48)**
LR χ^2^ (df)/F-statistics	**47.79(19)**	**F(19, 863) = 2.21**	**50.86 (19)**	F(19, 863) = 1.09
Pseudo/Adjusted R^2^	0.06	0.03	0.04	<0.001

Variables with a *p*-value <.05 are marked in bold.

Abbreviations: CI: Confidence interval, CP: Chronic pain, df: degrees of freedom, HoNos: The Health of the National Outcome Scales, OR: odds ratio, SMI: Severe mental illness.

^a^Odds ratio, 95% confidence intervals, LR χ^2^ (df) and pseudo-R^2^ are reported.

^b^Coefficient (β), 95% confidence intervals, F-statistics and adjusted R^2^ are reported.

## Discussion

To the best of our knowledge, this study is the first to examine the impact of diagnostic sequence on clinical and hospital admission outcomes in patients with comorbid CP and SMI. Key findings were that those diagnosed with CP before SMI (CP-SMI) experience significantly worse mental health outcomes. The CP-SMI group had higher odds of self-injury, substance misuse, depressive mood, and depression diagnoses. This finding is in line with existing evidence suggesting that delayed psychiatric recognition could exacerbates psychological distress,^31^ and can be understood within the biopsychosocial framework, which emphasises that physiological vulnerabilities (e.g. inflammation associated with both CP and SMI), psychological distress (e.g. low mood and maladaptive coping strategies) and social/system-level factors (such as care pathways and access to timely psychiatric support) interact to facilitate CP-SMI comorbidity.^32^

Underlying mechanisms explaining the disproportionate mental health burden observed in the CP-SMI group may be complex and multifaceted. Potential pathways may include CP as a significant risk factor for the development of mental health problems,^33^ or it underscores the detrimental effects of delayed psychiatric diagnosis. The phenomenon of diagnostic overshadowing, where CP dominates clinical attention, may contribute to this delay.^18,19^ CP’s somatic symptoms, such as joint or back pain, may overlap with or mask psychiatric symptoms, particularly depression, which shares features like fatigue and sleep disturbance.^34^ This is consistent with evidence that 75% of major depressive disorder patients report pain complaints, complicating timely psychiatric intervention.^20^ Moreover, culturally shaped expressions of distress can further obscure the identification of psychiatric symptoms, as somatic complaints may serve as the primary expression of psychological distress in some cultural contexts, such as in Arabian^35^ and Asian populations.^36^ Furthermore, the higher prevalence of depression diagnoses observed in the CP-SMI group suggests that psychiatric symptoms may only be recognized after significant deterioration, highlighting a reactive rather than proactive approach to mental health care in these patients. Delayed SMI recognition may exacerbate self-injury and substance misuse, as untreated psychological distress drives maladaptive coping mechanisms.^37^ Conversely, the SMI-CP group’s lower odds of these outcomes may reflect early or greater likelihood of psychiatric management and stabilization associated with a longer duration of SMI, in contrast of the CP-SMI group where their newly emerging psychiatric symptoms may be well less controlled, thereby supporting the protective role of timely SMI intervention.^15^

The lack of differences in hospital admissions between groups may reflect standardized care pathways in acute settings, where both CP and SMI trigger similar admission protocols.^16^ However, ethnic disparities, with higher psychiatric admission odds among Black and Mixed ethnicity patients, align with evidence of systemic inequalities in mental health care.^38^ Lower general admission odds for specific pain types (e.g. back, chest) may indicate outpatient management or varying healthcare-seeking behaviours, although the mechanisms underlying this remain unclear.^39^ The absence of differences in physical comorbidities suggests that diagnostic sequence primarily impacts mental health rather than physical disease burden, consistent with the focus on psychological outcomes in CP–SMI comorbidity.^8^ This finding may also reflect the under-recognition or under-recording of physical comorbidities once a SMI diagnosis is made, regardless of the temporal sequence of the two disorders. This result further underscores the role of diagnostic overshadowing and the barriers to care faced by patients with CP-SMI comorbidity.

Older age was protective against adverse mental health outcomes, potentially due to greater health literacy, social support, healthcare engagement, or higher socioeconomic status.^40^ This finding may also be explained by the fact that certain SMI diagnoses such as schizophrenia and bipolar disorder often have an early onset in young adulthood,^41^ meaning that first-onset incidence rates likely decline with increasing age. However, the increased prevalence of cognitive and physical comorbidities in older patients highlights their complex needs, necessitating tailored care.^42^ Female gender’s association with higher depression and anxiety odds aligns with broader epidemiological trends, while lower substance misuse odds may reflect gender-specific coping patterns.^23^

### Clinical and policy implications

These findings underscore the need to evaluate routine psychiatric screening aimed at preventing delayed SMI recognition at CP diagnosis. Validated tools like the Patient Health Questionnaire (PHQ-9) could identify early psychiatric symptoms in pain clinics.^43^ Given the modest efficacy of current pain interventions in SMI,^8^ robust evidence for an effective treatment specifically targeting CP remains limited. Integrated care models, which combine psychiatric, primary care, and pain specialists, are crucial for addressing CP–SMI comorbidity holistically, as recommended by recent reviews.^8,10^ Policy efforts should focus on reducing ethnic disparities and improving access to co-located services, particularly for marginalized populations.^17^

### Strengths and limitations

Strengths include a large, diverse cohort, comprehensive linked EHR data, and robust statistical adjustments, providing real-world insights into CP–SMI comorbidity. Limitations include the potential for underdiagnosis of CP in SMI patients, which limits generalizability.^44^ CP is widely recognised to be underdiagnosed among individuals with SMI,^45^ although the extent of this under-recording in this study population is difficult to quantify considering empirical estimates of underdiagnosis rate remain limited in current literature. To explore this indirectly, we compared CP identification via diagnostic codes and through analgesic prescriptions. In both CP-MH and MH-CP cohorts, a greater proportion of CP cases were identified using CP diagnostic codes (57% vs 66%, respectively) than through analgesic prescription alone, (18% vs 23%). While this pattern may suggest a slightly higher likelihood of CP being formally coded among individuals with SMI diagnosis, it does not necessarily imply better detection in this group. Instead, it may simply reflect the higher underlying prevalence of CP among individuals with SMI.^8^ This issue is further compounded by the possibility that CP experiences are under-recorded in primary care record. Recorded rates of SMI diagnoses in UK GP data appear broadly consistent with the incidence estimates from existing epidemiological studies,^46^ suggesting reasonably complete ascertainment in primary care records. However, the completeness of CP coding is less certain. Previous comparisons of pain recording between CRIS and LDN^47^ indicate discrepancies, with more patients with pain in general being identified in CRIS than LDN. This suggests potential under-recording or inconsistent coding practice of physical conditions, particularly CP, which may be managed symptomatically without always being formally coded. It is also plausible that SMI diagnoses are more consistently recorded due to an incentivised monitoring framework (i.e. Quality and Outcomes Framework) in UK primary care, whereas CP may be less systematically recorded. Although the extent of under-recording cannot be precisely estimated, incorporating analgesics records as a supplementary ascertainment strategy may have improved the capture of CP. Nevertheless, the under-recording of CP remains possible and should be considered when interpreting our findings. Low model R^2^ values suggest the presence of unmeasured confounders (e.g. symptom severity, social support). System-level factors (e.g. access to care) may influence outcomes, and the findings may not be generalizable to non-universal healthcare systems. The reliance on diagnostic codes and prescriptions may introduce misclassification, as pain may be undocumented or medications used for non-pain indications. Lastly, this study captured mental health comorbidities ever recorded within patients’ records, which may have included diagnoses made well before the onset of CP or SMI. This limits our ability to determine the temporal sequence of comorbidity and may overestimate the associations if pre-existing mental illness influenced the development or recognition of CP and/or SMI.

### Future research

Future studies should explore the mechanisms of diagnostic overshadowing through qualitative research, capturing the perspectives of both patients and providers. Longitudinal designs incorporating biological markers (e.g. inflammatory cytokines) could clarify causal pathways between CP and SMI.^12^ Randomized controlled trials should evaluate integrated interventions, such as cognitive behavioural therapy (CBT) or multidisciplinary care, to improve outcomes.^8^ Comparative studies across healthcare systems could elucidate structural barriers to care access, informing global policy.^10^

## Conclusion

This study provides novel evidence that patients diagnosed with CP before SMI face worse mental health outcomes. This could be due to delayed psychiatric intervention. Evaluating how routine psychiatric screening and integrated care at CP diagnosis may mitigate psychological distress, reduce healthcare burden, and address mental health inequalities is essential. These findings advocate for a paradigm shift toward multidisciplinary, biopsychosocial approaches to CP–SMI comorbidity, enhancing patient outcomes and quality of life.

## Supplemental material

Supplemental material - Diagnostic sequence of chronic pain and severe mental illness: Relationship with mental health and hospitalization outcomes Supplemental material for Diagnostic sequence of chronic pain and severe mental illness: Relationship with mental health and hospitalization outcomes by Ruimin Ma, Eugenia Romano, Mark Ashworth, Nilufar Mossaheb, Kerem Böge, Davy Vancampfort, Robert Stewart and Brendon Stubbs in British Journal of Pain.

## Data Availability

The data used in this study were obtained from routinely collected healthcare records and contain sensitive personal information. Due to data confidentiality and ethical restrictions, these data cannot be shared publicly. Access to the data may be available upon reasonable request to the corresponding author and with appropriate institutional and ethical approvals.*
