# Supplementary material for: Diagnostic sequence of chronic pain and severe mental illness: Relationship with mental health and hospitalization outcomes
Source: Br J Pain. 2026 May 28:20494637261451733. Online ahead of print. doi: 10.1177/20494637261451733 (PMC13219134; doi:10.1177/20494637261451733)
Supplement: Supplemental material - Diagnostic sequence of chronic pain and severe mental illness: Relationship with mental health and hospitalization outcomes [file sj-pdf-1-bjp-10.1177_20494637261451733.pdf]

Supplementary Table 1. Multivariable linear and logistic regression models for physical comorbidities, controlling for covariates

Predictors	**Number of physical comorbidities**	
	**Comorbidities developed after index date Coefficient (95% CI)**	**Comorbidities ever developed Coefficient (95% CI)**
**Cohorts**		
CP-SMI group	Reference	Reference
SMI-CP group	0.03 (-0.03, 0.09)	0.03 (-0.08, 0.14)
**SMI diagnosis**		
Schizophrenia	Reference	Reference
Bipolar	-0.06 (-0.13, 0.01)	-0.03 (-0.17, 0.11)
Depressive	**0.13 (0.03, 0.25)**	0.16 (-0.05, 0.37)
Unspecified SMI	-0.03 (-0.16, 0.10)	-0.01 (-0.26, 0.24)
**Age at index date**	**-0.004 (0.01, -0.00)**	**0.03 (0.03, 0.04)**
**Gender**		
Male	Reference	Reference
Female	0.02 (-0.04, 0.08)	-0.01 (-0.12, 0.10)
**Ethnicity**		
White	Reference	Reference
Black	**-0.08 (-0.14, -0.12)**	**0.22 (0.09, 0.34)**
Asian	**-0.14 (-0.26, -0.03)**	0.16 (-0.06, 0.39)
Mixed	-0.10 (-0.22, 0.03)	**0.31 (0.08, 0.55)**
Other	-0.04 (-0.19, 0.11)	-0.00 (-0.30, 0.29)
**Deprivation score**	0.00 (-0.00, 0.01)	0.00 (-0.00, 0.01)
**CP diagnosis**		
Abdominal pain	Reference	Reference
Back pain	-0.02 (-0.14, 0.10)	0.06 (-0.17, 0.29)
Chest pain	0.00 (-0.12, 0.13)	0.16 (-0.09, 0.40)
Facial pain	0.06 (-0.08, 0.21)	0.09 (-0.19, 0.38)
Generalised pain not localised/Pain unspecified	-0.01 (-0.10, 0.08)	0.14 (-0.04, 0.31)
Lower Limb pain	-0.27 (-0.13, 0.08)	0.07 (-0.13, 0.27)
Pelvic pain	-0.05 (-0.21, 0.11)	-0.07 (-0.38, 0.23)
Upper Limb pain	-0.00 (-0.18, 0.17)	0.19 (-0.14, 0.53)
**Number of physical comorbidities before index**	**0.31 (0.27, 0.34)**	
**F-statistics**	**F(19, 863)= 19.82**	**F(18, 864)=18.73**
**Adjusted R^2**	0.29	0.27

Variables with a p-value <0.05 are marked in bold.
Abbreviations:
CI: Confidence interval
CP: Chronic pain
df: degrees of freedom
HoNos: The Health of the National Outcome Scales
OR: odds ratio
SMI: Severe mental illness
